# Long-term leukocyte reconstitution in NSG mice transplanted with human cord blood hematopoietic stem and progenitor cells

**DOI:** 10.1186/s12865-017-0209-9

**Published:** 2017-05-30

**Authors:** Annette Audigé, Mary-Aude Rochat, Duo Li, Sandra Ivic, Audrey Fahrny, Christina K. S. Muller, Gustavo Gers-Huber, Renier Myburgh, Simon Bredl, Erika Schlaepfer, Alexandra U. Scherrer, Stefan P. Kuster, Roberto F. Speck

**Affiliations:** 1 Division of Infectious Diseases and Hospital Epidemiology, University Hospital of Zurich, University of Zurich, Raemistrasse 100, 8091 Zurich, Switzerland; 2 Institute of Medical Virology, University of Zurich, Zurich, Switzerland

**Keywords:** Hematopoiesis, Hematopoietic stem cells, Cord blood stem cell transplantation, Humanized mice, NSG mice

## Abstract

**Background:**

Humanized mice (hu mice) are based on the transplantation of hematopoietic stem and progenitor cells into immunodeficient mice and have become important pre-clinical models for biomedical research. However, data about their hematopoiesis over time are scarce. We therefore characterized leukocyte reconstitution in NSG mice, which were sublethally irradiated and transplanted with human cord blood-derived CD34+ cells at newborn age, longitudinally in peripheral blood and, for more detailed analyses, cross-sectionally in peripheral blood, spleen and bone marrow at different time points.

**Results:**

Human cell chimerism and absolute human cell count decreased between week 16 and 24 in the peripheral blood of hu mice, but were stable thereafter as assessed up to 32 weeks. Human cell chimerism in spleen and bone marrow was maintained over time. Notably, human cell chimerism in peripheral blood and spleen as well as bone marrow positively correlated with each other. Percentage of B cells decreased between week 16 and 24, whereas percentage of T cells increased; subsequently, they levelled off with T cells clearly predominating at week 32. Natural killer cells, monocytes and plasmacytoid dendritic cells (DCs) as well as CD1c + and CD141+ myeloid DCs were all present in hu mice. Proliferative responses of splenic T cells to stimulation were preserved over time. Importantly, the percentage of more primitive hematopoietic stem cells (HSCs) in bone marrow was maintained over time.

**Conclusions:**

Overall, leukocyte reconstitution was maintained up to 32 weeks post-transplantation in our hu NSG model, possibly explained by the maintenance of HSCs in the bone marrow. Notably, we observed great variation in multi-lineage hematopoietic reconstitution in hu mice that needs to be taken into account for the experimental design with hu mice.

**Electronic supplementary material:**

The online version of this article (doi:10.1186/s12865-017-0209-9) contains supplementary material, which is available to authorized users.

## Background

Humanized mice (hu mice) generated via the transplantation of human hematopoietic stem and progenitor cells (HSPCs), fetal tissues or mature immune cells into immunodeficient mice serve as small animal models for biomedical research [1]. Immunodeficiency is a pre-requisite for efficient engraftment. Various immunodeficient mouse strains are in use for the generation of hu mice. They all have in common a mutated Prkdc gene (encoding DNA-dependent protein kinase, catalytic subunit) or a targeted disruption in the Rag1/2 genes which result in a lack of functional T and B cells; in most cases, the IL2 receptor common gamma chain (IL2rg) is also disrupted, compromising the development of murine innate immune cells including nature killer (NK) cells [2–4].

The efficiency of engraftment is also dependent on a “do not eat me” signal triggered by the binding of CD47, which is ubiquitously expressed, to the signal regulatory protein alpha (SIRPα) receptor on phagocytic cells [5]. The mouse SIRPα allele on macrophages of Non-obese-diabetic (NOD) mice efficiently recognizes the human CD47 on engrafted cells and thus prevents their phagocytosis [6]. In contrast, the SIRPα allele of C57BL/6 mice does not bind to human CD47 and thus does not trigger this inhibitory axis, resulting in phagocytosis of xenotransplanted cells and lack of reconstitution of human hematopoiesis; the SIRPα allele of BALB/c mice moderately binds to human CD47, resulting in limited graft efficiency [7].

Strains of immunodeficient IL2rg^null^ mice on a NOD background that are commonly used by investigators include NOD.Cg-*Prkdc* ^*scid*^ *Il2rg* ^*tm1Sug*^ (abbreviated NOG) [2, 8], NOD.Cg-*Prkdc* ^*scid*^ *Il2rg* ^*tm1Wjll*^ (NSG) [3, 9], and NOD.Cg-*Rag1* ^*tm1Mom*^ *Il2rg* ^*tm1Wjl*^ */SzJ* (NRG) [10]. NOG and NSG mice both have a mutated Prkdc gene, whereas NRG mice have a targeted disruption in the Rag1 gene; NOG mice have a cytoplasmic truncation, and NSG mice a complete deletion of the IL2rg. Engraftment of human hematopoietic stem cells (HSCs) derived from umbilical cord blood is more efficient in NSG mice than NOG mice [11], but similar between NSG and NRG mice [12]. The difference in the overall engraftment between NOG and NSG mice is likely attributable to the presence of the IL2rg extracellular domain in the NOG mice [11]. Currently, the most widely used strain for generating hu mice is the NSG mouse.

In NSG mice, human cell chimerism was shown to be maintained up to 24 weeks post-transplantation; the number of mice used, however, was only three, making it difficult to draw any firm conclusions [9]. Only two studies reported hematopoietic cell reconstitution beyond 24 weeks post-transplantation; these studies used NRG mice [13] and BALB/c-*Rag2* ^*null*^ *Il2rg* ^*null*^ (BRG) mice [14] transplanted at newborn age with cord blood-derived cells. In NRG mice, lymphoid cells and monocytes remained stable in the peripheral blood for ~1 year [13], whereas in BRG mice, a decline of human cell chimerism from week 6 to week 40 in blood and bone marrow and after week 24 in spleen was noted [14]. Notably, hematopoietic cell reconstitution, especially the development of B and T cells, is dynamic, with B cells decreasing and T cells increasing during the first 3 to 4 months irrespective of the mouse strain [1, 13, 14].

The aim here was to assess whether leukocyte reconstitution in hu NSG mice is maintained beyond week 24 post-transplantation. We addressed this question by monitoring human cell chimerism, absolute human cell count and reconstitution of B and T cells longitudinally between week 16 and 32 in peripheral blood. We also did a more detailed analysis, including reconstitution of other hematopoietic cell populations such as NK cells and dendritic cells (DCs), cross-sectionally at week 16, 24, or 32 post-transplantation in peripheral blood, spleen and bone marrow. Engraftment of HSPCs and more primitive hematopoietic stem cells (HSCs) in bone marrow was also analyzed. We started our analyses at week 16 as leukocyte reconstitution in hu mice is, as mentioned above, dynamic until this time point post-transplantation. Our data support overall maintenance of leukocyte reconstitution up to 32 weeks post-transplantation in our hu NSG model, but also reveal high inter-animal variation in leukocyte subset reconstitution.

## Methods

### Humanized mice

Immunodeficient NOD.Cg-*Prkdc* ^*scid*^ *Il2rg* ^*tm1Wjll*^ (NSG) mice were obtained from The Jackson Laboratory or Charles River Laboratories. For reconstitution, newborn NSG mice were irradiated 1–2 days after birth with 1 Gy and subsequently injected intrahepatically (i.p.) with 1.74 ± 0.57 x 10^5^ (mean ± SD) CD34+ cells. CD34+ cells were isolated from human cord blood with immunomagnetic beads (CD34 MicroBead Kit; Miltenyi Biotec) with a purity of 92.8 ± 5.9% (*n* = 25) and cryopreserved in FBS and 10% DMSO until use. Procurement of human cord blood was approved by the ethical committee of the University of Zurich. Human cord blood was collected with informed written consent of the parents. All animal experiments were approved by the Cantonal Veterinary Office.

In some cases, mice presented progressive alopecia indicating graft versus host disease. Mice with alopecia were not included into experiments respectively mice showing symptoms of graft versus host disease during the monitoring period were excluded from analyses.

Mice originating from the same litter are referred to as a group of mice; a cohort of mice consisted of several groups of mice.

### Immunostaining and flow cytometry

Human cell chimerism and percentages of human cell populations were determined in peripheral blood, spleen and bone marrow at various time points. For absolute cells counts, peripheral blood was collected into EDTA tubes (BD Biosciences). White blood cell counts (WBC/ml) were determined with a COULTER® Ac · T diff™ Analyzer (Beckman Coulter) using 20 μl of peripheral blood diluted in buffer according to the manufacturer’s instructions. Otherwise, peripheral blood was collected from the tail vein into FACS tubes containing FACS buffer (PBS containing 2 mM EDTA, 2% FBS, 0.1% sodium azide), centrifuged and incubated with antibodies in FACS buffer for 20 min at room temperature (RT). Erythrocytes were lysed with BD FACS Lysing Solution (BD Biosciences) for 10 min at RT. Cells were washed with PBS containing 2 mM EDTA, resuspended in FACS buffer and kept at 4 °C until flow cytometric analysis.

Splenocytes were obtained by squeezing the spleens through a 70 μm nylon mesh. Bone marrow cells were obtained by cutting both ends of the femur and tibia, flushing the bone marrow with PBS containing 2% FBS and 2 mM EDTA, and passing the cells through a 70 μm nylon mesh. Erythrocytes in both spleen and bone marrow cell suspensions were lysed with ACK lysis buffer (Lonza) and were cryopreserved until use.

Cryopreserved bone marrow and spleen cells were thawed, counted and incubated with antibodies in FACS buffer for 20 min at 4 °C. Cells were washed with FACS buffer, fixed with 2% paraformaldehyde and kept at 4 °C until flow cytometry. Control staining included all antibodies except those against minor cell populations (e.g., NK cells and DCs). Mouse BD Fc Block™ was included for staining of bone marrow cells for HSPCs; PE/Cy7 Mouse IgG1 κ Isotype Ctrl Antibody was used as control for anti-CD141 PE-Cy7 antibody in staining of splenocytes.

For measuring cell proliferation and activation upon stimulation, splenocytes and human peripheral blood mononuclear cells (PBMCs) were incubated with Dynabeads® Human T-Activator CD3/CD28 (Life Technologies) at a bead-to-cell ratio of 1:1 for 72 h. Human PBMCs were isolated from buffy coats (local blood donation center in Zurich) by Lymphoprep™ (Alere Technologies AS) density gradient centrifugation. After removal of the beads, cells were incubated with antibodies against cell surface antigens (CD45, CD3, CD4, CD8, CD25) in FACS buffer for 20 min at 4 °C, washed with FACS buffer, fixed/permeabilized with BD Cytofix/Cytoperm™ (BD Biosciences) for 20 min at 4 °C, washed with 1× BD Perm/Wash solution, incubated with anti-Ki67 antibody in 1× BD Perm/Wash solution for 30 min at 4 °C, washed with 1× BD Perm/Wash solution, resuspended in FACS buffer and kept at 4 °C until flow cytometry analysis.

Antibodies (all specific for human, unless otherwise indicated) were purchased from BD Biosciences, BioLegend, Beckman Coulter or Miltenyi Biotec (Additional file 1: Table S1). Stained cells were acquired on a CyAn ADP flow cytometer (Beckman Coulter) and flow cytometry data were analyzed with FlowJo Version 7.2.5 or 10.0.8 (Tree Star). Doublets and cells with a low FSC/SSC profile were gated out.

### Immunoglobulin ELISA

Human IgM and IgG concentrations in plasma were determined using Human IgM and IgG ELISA Kit (Bethyl Laboratories), respectively, following the manufacturer’s instructions.

### Statistical analysis

For statistical analysis of longitudinal data between week 12 and 24 post-transplantation, we applied multilevel mixed-effects linear regression models with a random-intercept term at mice level, with a significance level of *p* < 0.05 using Stata® SE version 14.0 (Stata Corporation, College Station, TX). Paired t tests were used to compare week 16/24 or week 24/32, with a significance level of *p* < 0.05 using GraphPad prism 6 (GraphPad Software, Inc).

For statistical analysis of cross-sectional flow cytometry and immunoglobulin production data, groups of differentially aged mice were compared using one-way analysis of variance (ANOVA) followed by Tukey post-hoc test using GraphPad prism 6. Values in the text indicate mean ± SD. Bars in graphs in Figs. 3, 4, 5, 6, 7, 8, 9, 10, 11 and Additional file 2: Figure S3 indicate mean values. Paired t tests were used to compare the percentage of CD45+ cells between peripheral blood and spleen or bone marrow. A significance level of p < 0.05 (* *p* < 0.05, ** *p* ≤ 0.01, *** *p* ≤ 0.001) was used for all tests.

For sample size calculations, we used Sattertwaite’s t tests assuming unequal variances in order to provide an estimation of the sample sizes needed to detect a decline in the percentage of CD4+ T cells between 0 and 25% for different power assumptions at a two-sided α-level of 0.05. Stata® version 13.1 was used for sample size calculations.

## Results

### Human cell chimerism, absolute human cell count and reconstitution of B and T cells in peripheral blood

**Fig. 1:**
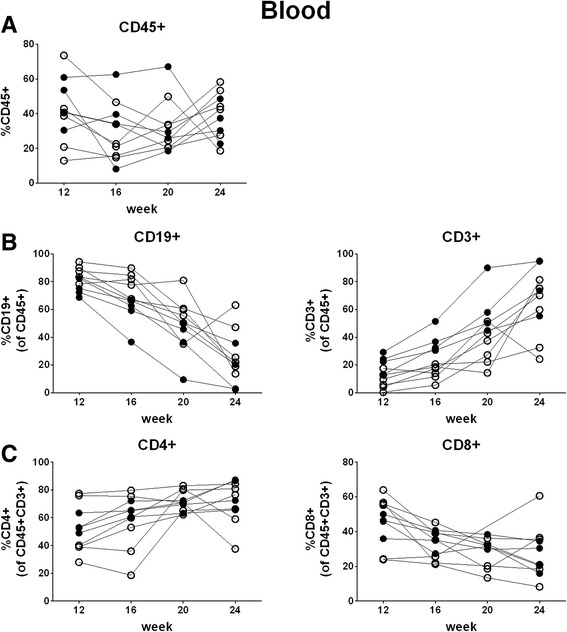
Longitudinal analysis of human cell chimerism and B and T cells in peripheral blood between week 12 and 24 post-transplantation. Human cell chimerism and percentages of B and T cells were assessed in peripheral blood longitudinally every four weeks between week 12 and 24 post-transplantation. Each symbol represents an individual mouse; data are derived from two groups of mice, which are indicated by different symbols (open/closed) (*n* **=** 10; number of mice per group: 4–6); each group of mice was transplanted with different cord blood-derived cells. **a** percentage of human cells (CD45+), **b** percentage of B cells (CD19+) and T cells (CD3+), **c** percentage of CD4+ and CD8+ cells within the T cell population

As most studies using hu mice are performed between week 12 and 24 weeks post-transplantation, we first monitored human cell chimerism and reconstitution of B and T cells in peripheral blood longitudinally every four weeks between week 12 and 24 after transplantation with cord blood-derived CD34+ cells in two groups of hu mice. Overall, human cell chimerism, as determined by the percentage of human CD45+ cells, showed inter- and intra-animal variation, but was not significantly declining over time (coefficient -0.8, 95% CI: -2.1 – 0.6, *p* = 0.27) (Fig. 1a). In contrast, the percentages of B and T cells changed over time, with a predominance of B cells at week 12 and a steady decrease thereafter up to week 24 post-transplantation (coefficient -4.7, 95% CI: -5.6 – -3.9, p < 0.001) and vice versa a low percentage of T cells at week 12 and subsequently a steady increase (coefficient 4.4, 95% CI: 3.5 – 5.3, *p* < 0.001) (Fig. 1b). Within the T cell population, the percentages of CD4+ and CD8+ T cells also changed over time (coefficient 1.8, 95% CI: 0.9–2.7 and -1.4, 95% CI: -2.2 – -0.7, *p* < 0.001 for both) in favor of CD4+ T cells (Fig. 1c). Thus, the B and T cell compartment is dynamic with a significant change of the ratio of B and T cells during the first 24 weeks post-transplantation.

**Fig. 2:**
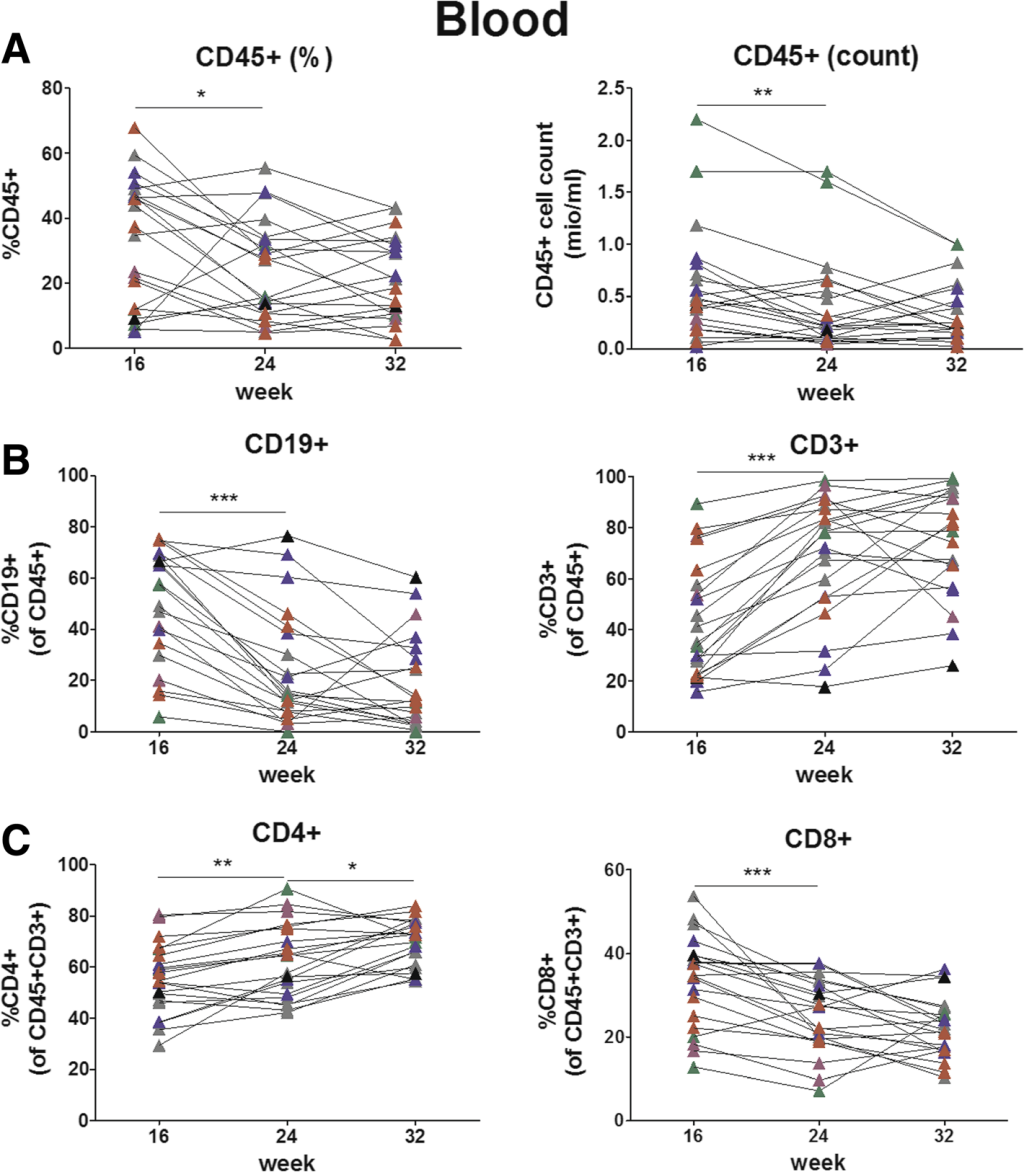
Longitudinal analysis of human cell chimerism, absolute human cell count and B and T cells in peripheral blood between week 16 and 32 post-transplantation. Human cell chimerism, absolute human cell count and percentages of B and T cells were assessed in peripheral blood longitudinally between week 16 and 32 post-transplantation. Each symbol represents an individual mouse; data are derived from 6 groups of mice (indicated by different colours) (*n* **=** 21; number of mice per group: 1–7); each group of mice was transplanted with different cord blood-derived cells. **a**: percentage and absolute count of human cells (CD45+), **b**: percentage of B cells (CD19+) and T cells (CD3+), **c**: percentage of CD4+ and CD8+ cells within the T cell population

To study hematopoietic cell reconstitution beyond 24 weeks post-transplantation, we monitored human cell chimerism, absolute human cell count and reconstitution of B and T cells in peripheral blood longitudinally between week 16 and 32 after transplantation in several groups of hu mice. Human cell chimerism, in percent (Fig. 2a, left and Table 1) as well as absolute cell numbers (Fig. 2a, right) slightly decreased between week 16 and 24 (*p* = 0.046, paired t test), but was stable between week 24 and 32. In contrast, lymphoid cell subsets showed substantial changes between week 16 and 24: B cells decreased and T cells increased (Fig. 2b). Since B and T cells are the two main lymphoid cell populations, B and T cell percentages are largely inversely proportionate. After week 24, the percentages of both subsets were stable. We also observed a dynamic pattern for CD4+ and CD8+ T cells: CD4+ T cells increased over time, predominantly between week 16 and 24, whereas CD8+ T cells decreased between week 16 and 24 (Fig. 2c). Thus, human cell chimerism, absolute human cell count and particularly reconstitution of B and T cells in peripheral blood are dynamic up to week 24 and then level off.

**Table 1:** Human cell chimerism and reconstitution of lymphocytes in peripheral blood (longitudinal)

	16 weeks	24 weeks	32 weeks
CD45+	33.5 ± 19.7%	24.4 ± 15.3%	21.8 ± 12.9%
B cells	49.5 ± 22.2%	24.6 ± 22.5%	18.8 ± 18.2%
T cells	43.6 ± 22.8%	69.3 ± 23.8%	74.1 ± 21.0%
CD4 T cells	55.5 ± 13.8%	62.5 ± 15.6%	69.4 ± 9.2%
CD8 T cells	33.5 ± 11.0%	25.1 ± 8.8%	22.3 ± 7.2%

Percentages are given as mean ± SD; *n* = 21

**Fig. 3:**
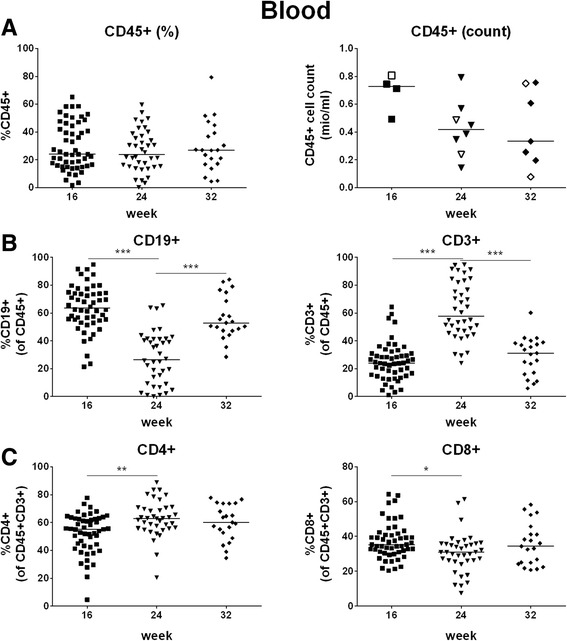
Cross-sectional analysis of human cell chimerism, absolute human cell count and B and T cells in peripheral blood at week 16, 24, or 32 post-transplantation. Human cell chimerism, absolute human cell count and percentages of B and T cells were assessed in peripheral blood cross-sectionally at week 16, 24 or 32 post-transplantation. **a** percentage and absolute count of human cells (CD45+), **b** percentage of B cells (CD19+) and T cells (CD3+), **c** percentage of CD4+ and CD8+ cells within the T cell population. A, left, B and C: each symbol represents an individual mouse; data are derived from multiple groups of mice per time point (number of groups: 16 weeks: *n* **=** 9, 24 weeks: *n* **=** 7, 32 weeks: *n* **=** 4; number of mice per group: 3–11); each group of mice was transplanted with cells derived from a different cord blood. A, right: each symbol represents an individual mouse; data are derived from two (16 and 32 weeks) or three (24 weeks) different groups of mice, which are indicated by different symbols (open/closed); each group was transplanted with cells derived from a different cord blood

We also determined relative and absolute human cell levels and reconstitution of B and T cells in peripheral blood in several groups of hu NSG mice cross-sectionally at week 16, 24, and 32. In essence, we obtained data similar to those from mice examined longitudinally (Fig. 3 and Table 2). We present these data here since the more detailed analyses, i.e., of additional hematopoietic cell populations in peripheral blood and of the reconstitution of leukocytes in organs (see below), require cross-sectional analyses.

**Table 2:** Human cell chimerism and reconstitution of lymphocytes in peripheral blood (cross-sectional)

	16 weeks	24 weeks	32 weeks
CD45+	29.7 ± 16.9%	26.4 ± 15.0%	29.5 ± 18.3%
B cells	63.7 ± 16.5%	27.7 ± 18.7%	55.6 ± 15.5%
T cells	25.0 ± 13.7%	61.9 ± 20.4%	29.1 ± 13.7%
CD4 T cells	52.3 ± 14.3%	62.9 ± 13.1%	60.2 ± 12.8%
CD8 T cells	37.4 ± 10.5%	30.6 ± 11.3%	35.6 ± 12.1%

Percentages are given as mean ± SD; *n* = 51 (16 weeks), 38 (week 24), 21 (week 32)

### Reconstitution of natural killer cells, monocytes and dendritic cells in peripheral blood

**Fig. 4:**
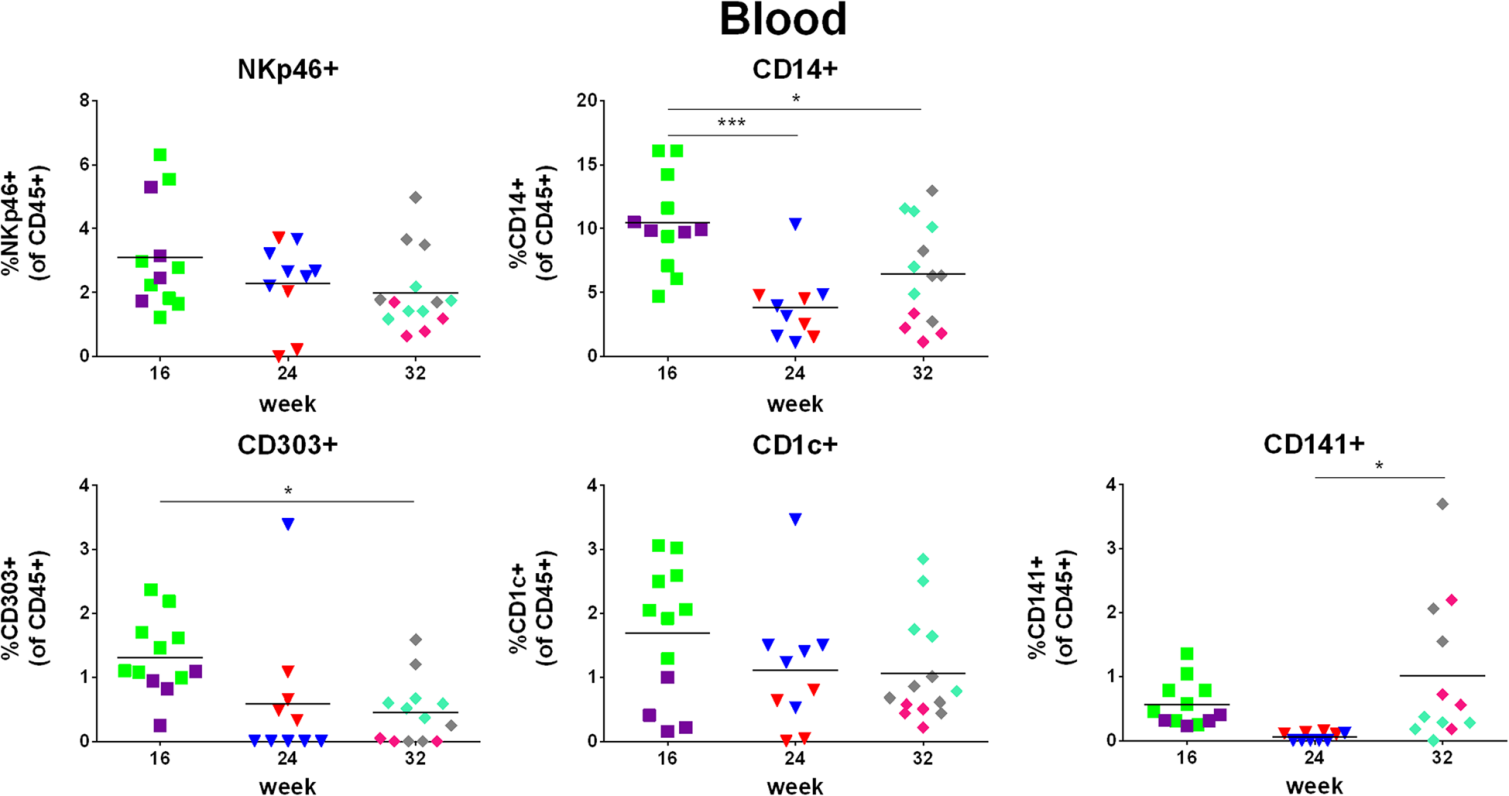
Reconstitution of NK cells, monocytes and DCs in peripheral blood. Reconstitution of NK cells, monocytes and DCs was assessed in peripheral blood of hu NSG mice at week 16, 24, or 32 post-transplantation. Gating strategy is shown in Additional file 7: Figure S1. Upper panel: percentage of NK cells (NKp46+) and monocytes (CD14+), lower panel: percentage of pDCs (CD303+), CD1c + mDCs (CD1c+) and CD141+ mDCs (CD141+). Each symbol represents an individual mouse; data are derived from two (16 and 24 weeks) or three (32 weeks) different groups of mice, which are indicated by different colours; each group was transplanted with cells derived from a different cord blood

We detected NK cells in 35/36 (97%), monocytes in 36/36 (100%), plasmacytoid DCs (pDCs) in 27/36 (75%), CD1c + myeloid DCs (mDCs) in 35/36 (97%), and CD141+ mDCs in 30/36 (83%) hu mice in the peripheral blood (Fig. 4 and Table 3). The percentage of NK cells remained stable over 32 weeks. Monocytes were less frequent at week 24 and 32 than week 16. The DC compartment gave a mixed picture: pDCs were less frequent at week 32 than week 16, CD1c + mDCs remained stable over time, and CD141c + DCs were more frequent at week 32 than week 24.

**Table 3:** Reconstitution of NK cells, monocytes and DCs in peripheral blood

	16 weeks	24 weeks	32 weeks
NK cells	3.10 ± 1.69%	2.28 ± 1.28%	1.99 ± 1.22%
Monocytes	10.5 ± 3.64%	3.83 ± 2.68%	6.44 ± 3.95%
pDCs	1.30 ± 0.60%	0.59 ± 1.05%	0.45 ± 0.50%
CD1c + mDCs	1.69 ± 1.06%	1.11 ± 1.00%	1.06 ± 0.81%
CD141+ mDCs	0.57 ± 0.36%	0.06 ± 0.07%	1.01 ± 1.14%

Percentages are given as mean ± SD; *n* = 12 (16 weeks), 10 (week 24), 14 (week 32)

### Hematopoietic cell reconstitution in spleen

**Fig. 5:**
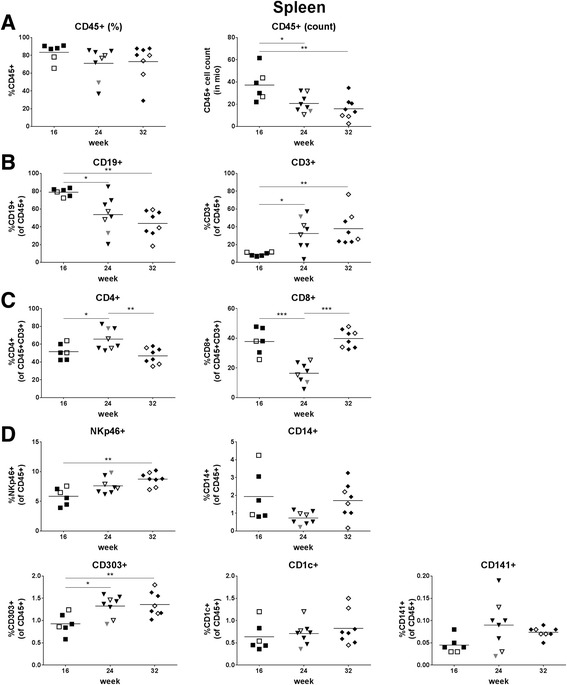
Leukocyte reconstitution in spleen**.** Human cell chimerism, absolute human cell count and reconstitution of lymphocytes, monocytes, and DCs were assessed in spleen at week 16, 24, or 32 post-transplantation. **a** percentage and absolute count of human cells (CD45+), **b** percentage of B cells (CD19+), and T cells (CD3+), **c** percentage of CD4+ and CD8+ T cells within the T cell population, **d** percentage of NK cells (NKp46+) and monocytes (CD14+), pDCs (CD303+), CD1c + mDCs (CD1c+) and CD141+ mDCs (CD141+). Each symbol represents an individual mouse; data are derived from two (16 and 32 weeks) or three (24 weeks) different groups of mice, which are indicated by different symbols (open/closed); each group was transplanted with cells derived from a different cord blood

Human cell chimerism in the spleen was on average >70% (Fig. 5a, left and Table 4) and thus much higher than in the peripheral blood (Additional file 3: Figure S2A, left); human cell chimerism in peripheral blood and spleen positively correlated with each other (*p* = 0.039) (Additional file 3: Figure S2A, right). Human cell chimerism in the spleen was similar between mice sacrificed at week 16, 24, or 32 post-transplantation (Fig. 5a, left). In contrast, we observed a time-dependent decrease of the absolute number of CD45+ cells (Fig. 5a, right). The analyses of B and T cells in the spleen essentially reflected the results obtained in the peripheral blood: we observed the highest percentage of B cells at week 16 and vice versa the lowest percentage of T cells at that time point; these percentages levelled off between week 24 and 32 (Fig. 5b and Table 4). Notably, CD4+ and CD8+ T cells showed a peak and nadir, respectively at week 24 (Fig. 5c). Regarding NK cells, monocytes, and DCs in spleen, there was a major difference to peripheral blood: these cell populations were present in the spleens of all hu mice analyzed and none of them declined over time (Fig. 5d). In line with these data, the percentage of total myeloid cells as defined by the CD33 marker was similar between the mice irrespective of their age (Additional file 2: Figure S3, left). Gating strategy for assessment of myeloid cell reconstitution in spleen is shown in Additional file 4: Figure S4A.

**Table 4:** Hematopoietic cell reconstitution in spleen

	16 weeks	24 weeks	32 weeks
CD45+	83.3 ± 9.93%	71.0 ± 18.2%	72.9 ± 20.1%
B cells	78.8 ± 4.42%	53.7 ± 20.5%	43.7 ± 14.7%
Mature	63.8 ± 3.63%	69.4 ± 12.3%	61.7 ± 11.8%
Memory	0.53 ± 0.26%	3.12 ± 2.24%	4.86 ± 1.61%
T cells	9.27 ± 2.14%	32.5 ± 18.0%	37.9 ± 19.0%
CD4 T cells	51.6 ± 8.89%	65.7 ± 12.0%	46.9 ± 8.77%
Memory	52.7 ± 9.87%	77.1 ± 14.0%	54.7 ± 26.4%
CD8 T cells	37.8 ± 8.75%	25.4 ± 19.2%	58.4 ± 32.3%
Memory	24.4 ± 4.71%	59.1 ± 22.8%	34.4 ± 30.8%
NK cells	5.85 ± 1.46%	7.61 ± 1.34%	8.74 ± 1.13%
Monocytes	1.94 ± 1.42%	0.73 ± 0.36%	1.71 ± 0.97%
pDCs	6.79 ± 1.92%	9.71 ± 1.97%	9.83 ± 1.28%
CD1c + mDCs	0.64 ± 0.32%	0.71 ± 0.25%	0.83 ± 0.37%
CD141+ mDCs	0.05 ± 0.02%	0.05 ± 0.06%	0.07 ± 0.01%

Percentages are given as mean ± SD; *n* = 6 (16 weeks), 8 (week 24), 8 (week 32)

### T cell differentiation and responsiveness in spleen

**Fig. 6:**
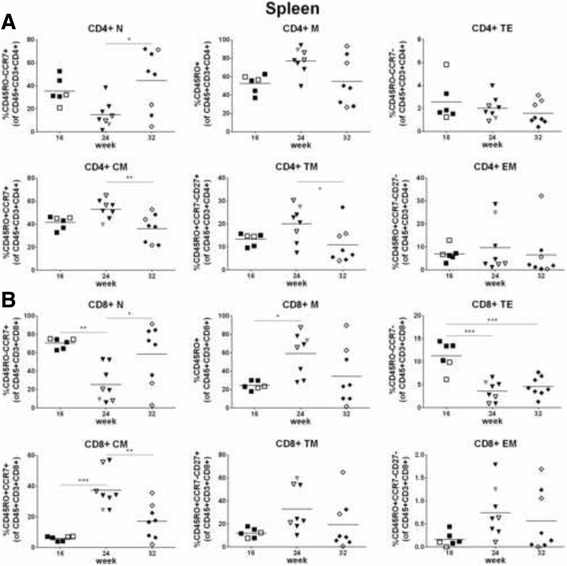
Differentiation of T cells in spleen. CD4+ (**a**) and CD8+ (**b**) T cells in spleen were analyzed for differentiation. Gating strategy is shown in Additional file 5: Figure S5A. T cell differentiation was defined as follows: naïve (N) = CD45RO-CCR7+, memory (M) = CD45RO+, terminal effector (TE) CD45RO-CCR7-, central memory (CM) = CD45RO + CCR7+, transitional memory (TM) = CD45RO + CCR7-CD27+, effector memory (EM) = CD45RO + CCR7-CD27-. Symbols: see Fig. 5

The differentiation profiles of CD4+ and CD8+ T cells were very similar: naïve cells and central memory cells within the memory cell population constituted the most frequent cell populations over time (Fig. 6). All kinds of differentiated T cells, including effector, transitional memory and effector memory cells, were present in the spleen. We observed a number of statistically significant differences when comparing the percentages of all cell subsets at the various time points, but without an obvious pattern (Fig. 6). Gating strategy for the analysis of T cell differentiation is shown in Additional file 5: Figure S5A.

**Fig. 7:**
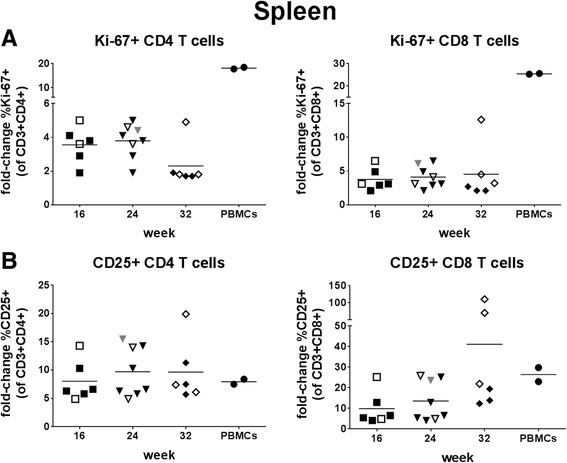
Functional characterization of T cells in spleen. Splenocytes and human PBMCs from two donors were stimulated with Dynabeads® Human T-Activator CD3/CD28 for 72 h and analyzed for proliferation (percentage of Ki67+ cells; **a**) and activation (percentage of CD25+ cells; **b**) of CD4 (left) and CD8 (right) T cells. Data are presented as fold-change relative to unstimulated cells. For each PBMC control, the mean fold-change from two experiments is shown. Symbols: see Fig. 5

Proliferation (Ki-67) and activation (CD25) of CD4+ and CD8+ T cells in spleen were similarly enhanced at all three time points post-transplantation upon stimulation with CD3/CD28 beads (Fig. 7). The proliferative response of splenic T cells from humanized mice was lower than that of human PBMCs, while their activation upon stimulation was similar to that of human PBMCs (Fig. 7).

### B cell maturation/differentiation in spleen

**Fig. 8:**
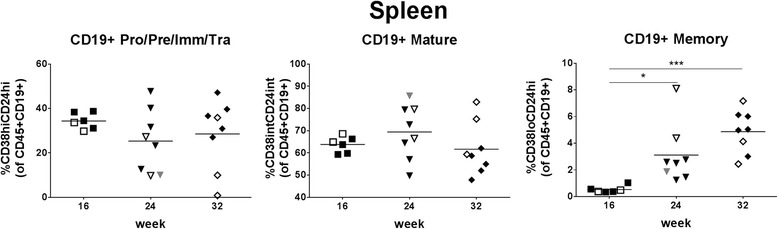
Maturation/differentiation of B cells in spleen. B cells in spleen were analyzed for maturation. Gating strategy is shown in Additional file 5: Figure S5B. B cell subpopulations were defined as follows: Pro/Pre/Imm/Tra (pro-B, pre-B, immature, transitional B cells) = CD38hiCD24hi, mature = CD38intCD24int, memory = CD38loCD24hi

The majority of B cells at ≥16 weeks post-transplantation were mature and the percentage of memory B cell increased over time (Fig. 8 and Table 4). Gating strategy for analysis of B cell maturation/differentiation is shown in Additional file 5: Figure S5B.

### Immunoglobulin production

**Fig. 9:**
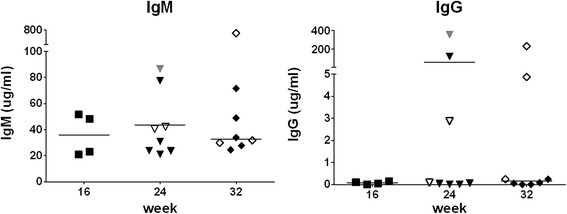
Production of immunoglobulins. Human IgM and IgG concentrations were determined in plasma (of the same mice as those data for which are shown in Figs. 5, 6, 7, 8). Symbols: see Fig. 5

IgM was consistently detected in all mice analyzed at a level of >20 μg/ml (Fig. 9). In contrast, IgG levels were <1 μg/ml in most animals. In five mice, IgG levels were >1 μg/ml and showed a high variability. IgM or IgG levels were similar between week 16, 24, and 32 post-transplantation.

### Leukocyte reconstitution and engraftment of HSPCs/HSCs in bone marrow

**Fig. 10:**
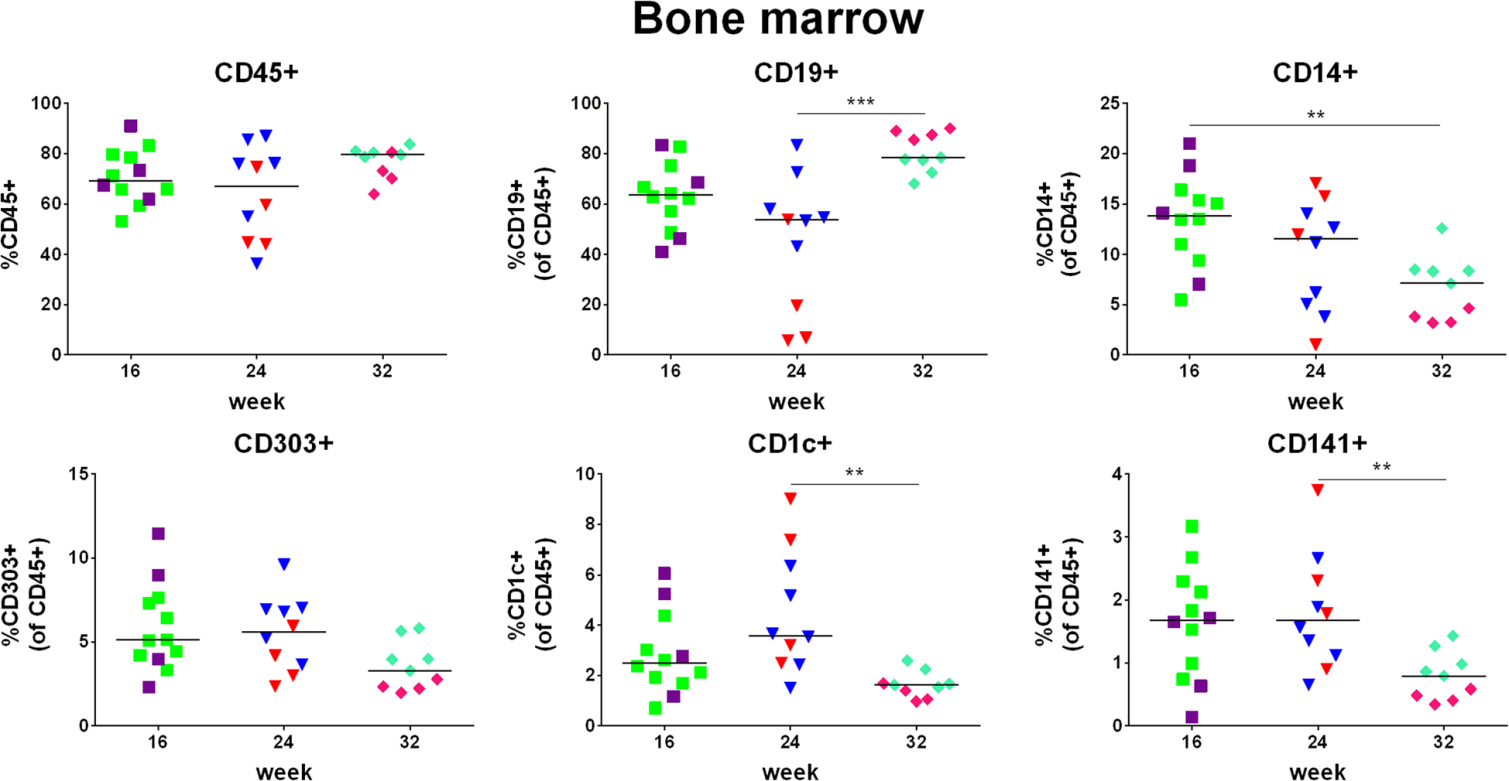
Leukocyte reconstitution in bone marrow. Human cell chimerism and percentages of B cells, monocytes and DCs were assessed in bone marrow (of the same mice as those data for which data are shown in Fig. 4) at week 16, 24, or 32 post-transplantation. Upper panel: percentage of human (CD45+) cells, B cells (CD19+), and monocytes (CD14+), lower panel: percentage of pDCs (CD303+), CD1c + mDCs (CD1c+) and CD141+ mDCs (CD141+). Symbols: see Fig. 4

Human cell chimerism (percentage of human CD45+ cells) in bone marrow was on average >64% irrespective of the time point post-transplantation when the mice were sacrificed (Fig. 10, upper panel, left and Table 5) and positively correlated with the one in the peripheral blood (*p* = 0.0008) (Additional file 3: Figure S2A, right). Furthermore, the percentage of CD34+ cells in the bone marrow significantly correlated with the percentage of human CD45+ cells in the peripheral blood (*r* = 0.359, *p* = 0.047; data not shown).

**Table 5:** Hematopoietic cell reconstitution and engraftment of HSPCs in bone marrow

	16 weeks	24 weeks	32 weeks
CD45+	70.9 ± 10.9%	63.9 ± 18.4%	76.8 ± 6.44%
B cells	63.2 ± 13.5%	45.1 ± 26.4%	80.7 ± 7.72%
Monocytes	13.4 ± 4.55%	9.85 ± 5.47%	6.64 ± 3.16%
DCs	10.3 ± 4.69%	11.7 ± 4.32%	5.98 ± 2.29%
CD34+	10.8 ± 2.70%	3.98 ± 2.57%	7.60 ± 1.82%
CD38-	1.26 ± 0.42%	0.84 ± 0.22%	0.84 ± 0.46%
CD90+	0.09 ± 0.05%	0.07 ± 0.04%	0.07 ± 0.05%

Percentages are given as mean ± SD; *n* = 12 (16 weeks), 10 (week 24), 9 (week 32)

Within the same animals, human cell chimerism and percentages of B cells, monocytes and DCs (Fig. 10 and Table 5) were significantly higher in bone marrow than in the peripheral blood (Additional file 3: Figure S2B, left). B cells constituted the cell population with the highest percentage in the bone marrow (Fig. 10, upper panel, middle and Table 5). At week 32, monocytes and mDCs showed a decrease in percentage as compared to the previous time points analyzed (Fig. 10, upper panel, right, and lower panel). However, the percentage of total myeloid cells in the bone marrow was similar between the different time points post-transplantation (Additional file 2: Figure S3, right). Gating strategy for assessment of myeloid cell reconstitution in bone marrow is shown in Additional file 4: Figure S4B. The discrepancy of decreasing monocytes and mDCs while overall myeloid cells remained stable over time might be explained by the presence of a variety of other myeloid cells not analyzed.

**Fig. 11:**
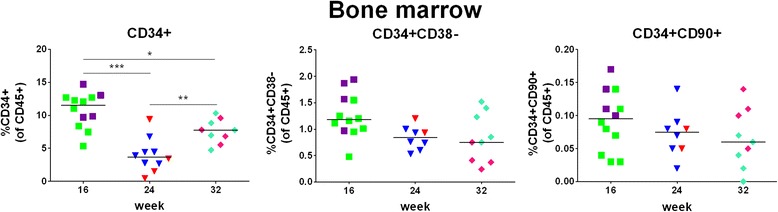
Engraftment of HSPCs/HSCs in bone marrow. Bone marrow of hu mice (same as those data for which are shown in Fig. 4) was analyzed at week 16, 24, or 32 post-transplantation for the percentages of total HSPCs (CD34+) among human CD45+ cells (left) and of more primitive cells (CD38- or CD90+; middle and right) among human CD45+ cells. Gating strategy is shown in Additional file 6: Figure S6. Symbols: see Fig. 4

We observed a decrease of the total CD34+ cells, including stem and progenitor cells, between week 16 and 24 (Fig. 11, left and Table 5). The percentages of the more primitive cells, as defined by CD34 + CD38- or CD34 + CD90+, were very low (Fig. 11, middle and right), but, importantly, remained in a similar range during the entire observation period. Gating strategy for analysis of engraftment of HSPCs/HSCs in bone marrow is shown in Additional file 6: Figure S6.

### Impact of inter-animal variation on design of experimental studies using hu mice

**Fig. 12:**
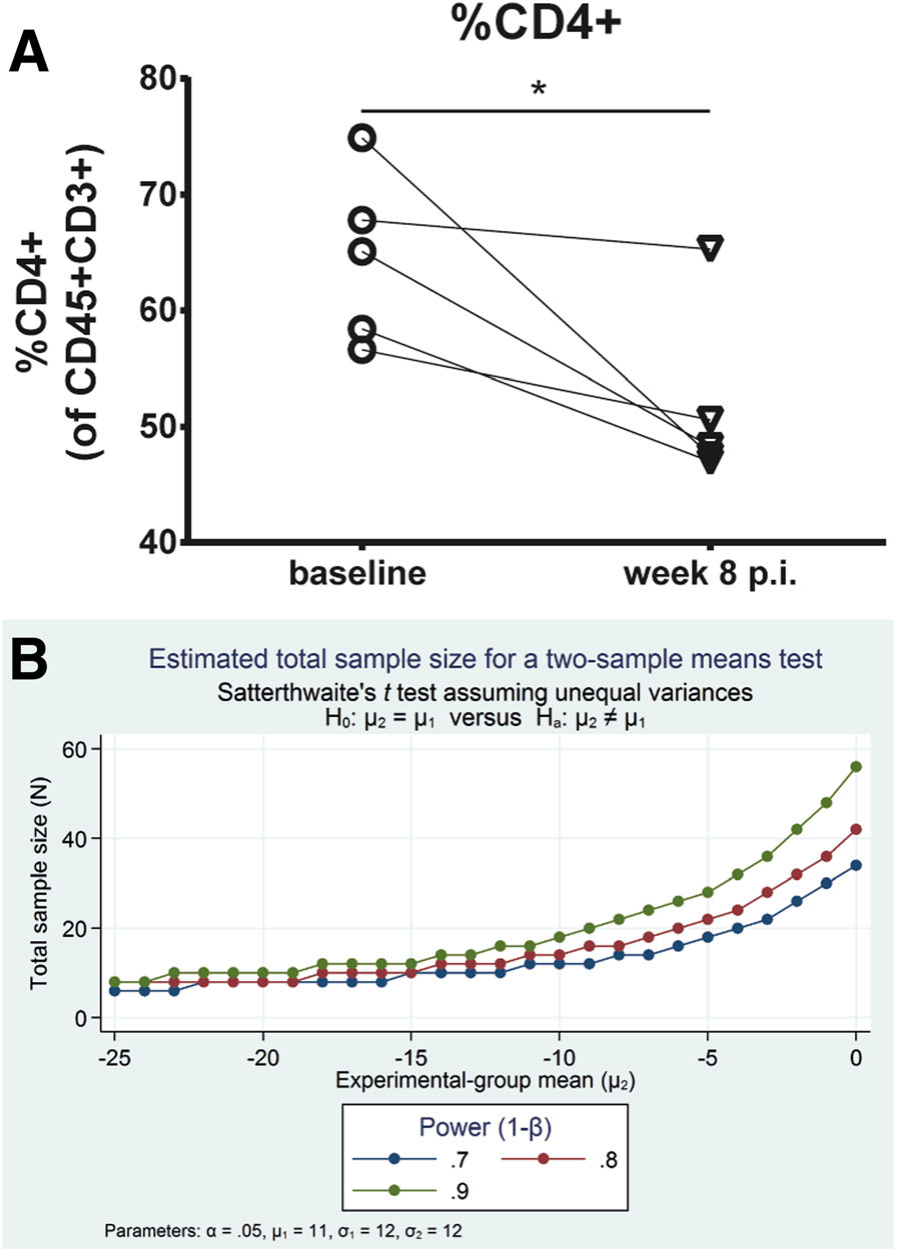
Example for estimated sample sizes required for animal experimentation. **a**: percentage of CD4+ T cells (of CD45 + CD3+ cells) in hu NSG mice was determined before (baseline) and at week 8 after infection with the R4-tropic HIV strain NL4-3. **b**: figure depicts total sample sizes required to detect a decline in the percentage of CD4+ T cells between 0% and 25% for different power assumptions. Calculations were based on a mean increase in the percentage of CD4+ T cells of 11.7% (SD: 12.4%) between week 16 and week 24 post-transplantation

Multi-lineage hematopoietic reconstitution showed great inter-animal variation in our hu NSG mice. This is of great importance for the design of experimental studies using hu mice. An example is the estimation of the number of mice required to monitor preservation of CD4+ T cells upon anti-HIV treatment in HIV-infected hu mice. Infection of hu mice with HIV strains, such as the CXCR4-tropic strain NL4-3, usually results in progressive CD4+ T cell depletion similarly to HIV-infected individuals. As shown for a group of HIV-infected mice, the average percentage of CD4+ T cells decreases by 13% during the first two months post-infection (p.i.) (Fig. 12a). In uninfected hu NSG mice, the average percentage of CD4+ T cells increases by 12% between week 16 and 24 post-transplantation (Fig. 2c, left). If the hypothesis is that the compound/drug preserves CD4+ T cells completely upon HIV infection, a total number of 40 mice is required for a statistical power of 0.8 (Fig. 12b).

## Discussion

Data on long-term multi-lineage hematopoiesis in hu mice are rather scarce. For future studies, detailed characterization is highly desirable, and thus, we filled this gap by examining multi-lineage hematopoiesis of hu NSG mice over time. The hu mice were generated using cord blood-derived CD34+ cells transplanted shortly after birth. We found that 1) human cell chimerism decreased in peripheral blood up to week 24, but thereafter levelled off until week 32; chimerism remained stable in spleen and bone marrow; absolute numbers decreased in peripheral blood up to week 24, and in spleen over the entire observation time; 2) lymphocyte cell subsets showed a dynamic picture with an initial decline of the percentage of B cells between week 16 and 24 and levelling off after week 24; the percentage of T cells behaved in an opposite manner with a clear predominance of T cells over B cells after week 24; 3) in the B cell compartment, we observed an increase of memory cells over time, which however did not translate into a progressive increase of IgG production; 4) we found a preserved proliferation response of T cells to stimulation over time; 5) other immune cells studied, i.e., NK cells, monocytes and DCs, were present over the entire observation period; 6) the level of multi-lineage hematopoietic reconstitution greatly varied between animals; and 7) the percentage of HSCs in bone marrow was stable over time and likely the basis for the long-term multi-lineage hematopoiesis observed. These data validate the hu mouse model for its use in biomedical research. The rather large variability needs to be taken into account when working with hu mice.

We report here that human cell chimerism was maintained over 32 weeks post-transplantation in hu NSG mice, extending previous data showing the preservation of multi-lineage hematopoiesis up to 24 weeks post-transplantation in NSG mice [9]. Consistent with our data, hu NRG mice transplanted i.p. with cord blood-derived CD34+ cells showed reconstitution of B and T cells and monocytes in peripheral blood for ~1 year [13]. In contrast, BRG mice transplanted i.p. or intravenously at newborn age with cord blood-derived CD34+ cells and T cell-depleted CD34- “support” cells showed a progressive decline of human cell chimerism [14]. The preserved multi-lineage hematopoiesis we observed in the NSG model as well as in the NRG model might be explained by the NOD background of the mice and its ability to promote the “do not eat me signal”. As mentioned above, BRG mice have a BALB/c background, which has a SIRPα allele poorly recognizing the human CD47 [7].

Blood human cell chimerism showed intra-animal variation over time. The reason for these fluctuations is unknown, but might relate to the bleeding method. Non-uniform warming of mice prior to tail bleeding can result in alterations in blood parameters, particularly of total white blood cells [15].

The predominance of B cells over T cells early after transplantation of HSPCs and its reversal later at/up to 12 to 16 weeks post-transplantation is a well-known phenomenon in hu mice [1]. We do not see this phenomenon in adult patients following allogeneic stem cell transplantation – both B and T cells show a protracted immune reconstitution with cell recovery in the 2^nd^ year after transplantation [16]. Our longitudinal data showing the dynamic of B and T cell development clearly speak in favor of a preserved multi-lineage hematopoiesis and that the human immune system in hu mice needs a rather long time to reach a plateau. In the NRG model, peripheral T cells similarly increased over time with subsequent levelling off at a high percentage at ~5 months lasting for more than a year; B cells showed the opposite pattern [13]. The same holds true for hu BRG mice, at least for the time frame reported, i.e., up to week 24 after transplantation [14].

The longitudinal and cross-sectional data of the reconstitution of B and T cells do not fully correspond to each other. We explain the discrepancy by the substantial variability in the reconstitution of T cells and associated decline in B cells between groups of hu mice, or even individual mice. Let’s remind that a group of mice is defined by the transplantation with the same donor blood.

The majority of B cells in the spleen in our model were mature over the entire observation period. Previous studies with hu mice on different mouse backgrounds reported a predominantly immature B cell phenotype [14, 17, 18]. Possible explanations for this discrepancy include differences in the mouse background, the age of the mice at the time point of analysis, and/or the markers used to determine maturation. Virtually all splenic mature B cells were in a naïve state 16 weeks post-transplantation consistent with data from Watanabe et al. [18]. We noted a subtle but statistically significant progressive increase of memory B cells over time in the spleen. The B cell compartment consistently produced human IgM in the hu NSG mice, but only rarely human IgG, indicating lack of Ig class switching. The IgM levels in the hu mice were in a similar range as in men based on the reference values provided by the manufacturer. The data concerning Ig class switching in hu mice are controversial. IgG could not be detected at week 12 post-transplantation in one study [17] and only in about half of animals in another study [19]. In contrast, Traggiai et al. detected IgG, albeit to various degrees, in older hu mice [4] and Lang et al. showed increasing levels of IgG between week 16 and 24 post-transplantation [14], indicating class switching. Again, we explain these divergent results by the different mouse strains and/or protocols used for generating the hu mice. Differences in the hygiene in the animal care facilities might be another factor to be considered.

The memory phenotype was strongly represented in the CD4+ as well as in the CD8+ T cell compartment in the spleen. In the NRG model discussed above, the memory CD4+ and CD8+ T cells, i.e., CD45RO+ cells, continually increased over time and were the predominant T cell subset at later time points in the peripheral blood [13]. Notably, they lost the CD28 cell surface marker pointing to a senescent state [13]. While we have not formally assessed senescence, stimulation-induced proliferation and activation did not differ at the various time points post-transplantation analyzed.

Crucially, other kinds of immune cells analyzed, including NK cells, monocytes, and DCs, were also present in the hu NSG mice over the entire observation period. Overall, their percentages at week 24 post-transplantation (when multi-lineage hematopoiesis levels off) were within the range of normal percentages of these cell types in human peripheral blood (NK cells: 1 − 6%, monocytes: 2 – 12%, DCs: 0.3 – 0.9% of leukocytes in human peripheral blood). In contrast, the percentages of B and T cells are much higher in our model and most other hu mouse models than in human peripheral blood (B cells: 1 – 7%, T cells: 7 – 24% of leukocytes in human peripheral blood). In human peripheral blood, myeloid cells predominate over lymphocytes (53 – 86 vs 14 – 47% of leukocytes, respectively), whereas in hu mice, human myeloid cells rarely exceed 5–10% of leukocytes [1]. Indeed, the percentage of myeloid (CD33+) cells in the spleens of our hu mice was on average 4% at 16, 24 and 32 weeks post-transplantation. This percentage was, however, much higher in the bone marrow, which can be explained by the fact that CD33 is also expressed on myeloid progenitors. An exception to the low percentage of myeloid cells in hu mice are the more recently developed strains MITRG and MISTRG, in which the percentage of these cells closely resembles that in humans [20].

Sustained multi-lineage hematopoiesis requires the maintenance of HSPCs in the bone marrow, at the best, of the most primitive cells. We found that the percentage of CD34+ HSPCs was lower at later time points but overall well preserved. Furthermore, the more primitive cells (CD34 + CD38- or CD34 + CD90+) were preserved over the entire period. We believe that this preservation contributes to the superior multi-lineage hematopoiesis in hu NSG mice as compared to other hu mouse models. Indeed, BRG mice transplanted with fetal liver-derived sorted CD34 + CD38- cells experienced a decrease of the percentage of CD34 + CD38- cells already at 6 weeks of age [21].

Multi-lineage hematopoietic reconstitution varied widely between animals in our hu NSG mouse model. This variation needs to be considered when designing experiments with hu mice, i.e., we need to integrate the dynamic nature of the lymphoid system into our power calculation as illustrated by the example for testing a compound’s protective activity against HIV-associated CD4+ T cell loss. Other estimations of sample sizes can be made based on our data sets. Thus, our study provides an important basis for the design of future experimental work using hu mice.

Notably, the NSG mouse is the most widely used strain for humanization. Novel strains are constantly being developed, mainly with knock-ins of human cytokines, or even knock-out of distinct genes for better engraftment. For example, derivatives of the NSG strain include NSG-SGM3 (or NSGS) mice, which express human stem cell factor, granulocyte-macrophage colony-stimulating factor, and interleukin-3 [22], and NSGW41 mice, which have a loss-of-function KIT receptor [23]. Human cell development in NSGS mice is skewed toward regulatory T cells, highlighting the importance of adequate spatiotemporal expression of human cytokines in xenotransplantation models [22]. NSGW41 mice support improved human erythropoiesis and platelet formation compared with irradiated NSG mice [24]. A characterization as we did here for NSG mice is necessary for each novel mouse model introduced into biomedical research.

## Conclusions

In summary, NSG mice transplanted with cord blood-derived CD34+ cells present preserved multi-lineage hematopoiesis up to 32 weeks. The considerable inter-animal variation and dynamic developmental pattern of various cell subtypes represent major challenges for the experimental design of experiments using hu mice.

## Additional files

Additional file 1: Table S1. Antibodies used for flow cytometry. (DOCX 40 kb)

Additional file 2: Figure S3. Myeloid cell reconstitution in spleen and bone marrow. Percentage of myeloid cells (CD33+) in spleen (A) and bone marrow (B). Gating strategy is shown in Additional file 4: Figure S4. Each symbol represents an individual mouse; for each time point post-transplantation, data are derived from two different groups of mice, which are indicated by different symbols (spleen: open/closed, bone marrow: different colors); each group was transplanted with cells derived from a different cord blood. (TIF 104 kb)

Additional file 3: Figure S2. Comparison of human cell chimerism between peripheral blood and spleen or bone marrow. Paired t test (left) and correlation (right) analyses were performed of human cell chimerism in peripheral blood and spleen (A) or bone marrow (B) (TIF 9278 kb)

Additional file 4: Figure S4. Gating strategy for assessment of myeloid cell reconstitution in spleen and bone marrow. Representative examples for the assessment of myeloid cell (CD33+) reconstitution in spleen (A) and bone marrow (B) are shown. (TIF 30248 kb)

Additional file 5: Figure S5. Gating strategy for assessment of T cell differentiation and B cell maturation/differentiation in spleen. Representative examples for the assessment of T cell differentiation (A) and B cell maturation/differentiation (B) are shown. (TIF 85813 kb)

Additional file 6: Figure S6. Gating strategy for assessment of engraftment of HSPCs in bone marrow. A representative example for the assessment of engraftment of HSPCs in bone marrow is shown. Percentages of cell populations were determined as follows: Total HSPCs: %CD34+ cells (of CD45+ cells), more primitive cells: %CD38- or CD90+ cells (of CD45 + CD34+ or CD45+ cells). (TIF 5264 kb)

Additional file 7: Figure S1. Gating strategy for assessment of human cell chimerism and reconstitution of leukocytes in peripheral blood. A representative example for the assessment of human cell chimerism and leukocyte reconstitution is shown. A: chimerism: %CD45+ cells (of live cells), B cells: %CD19+ cells (of CD45+ cells), monocytes: %CD14+ (of CD45+ cells), pDCs: %CD303+ cells (of CD45+ cells), CD1c + mDCs: %CD19-CD1c + cells (of CD45+ cells), CD141+ mDCs: %CD14-CD141+ cells (of CD45+ cells); B: T cells: %CD3+ cells (of CD45+ cells), CD4 T cells: %CD4+ cells (of CD45 + CD3+ cells), CD8 T cells: %CD8+ cells (of CD45 + CD3+ cells), NK cells: %CD3-NKp46+ (of CD45+ cells). (TIF 21813 kb)

## Abbreviations

hu mice: humanized mice
HSPCs: hematopoietic stem and progenitor cells
IL2rg: IL2 receptor common gamma chain
NK: natural killer
SIRPα: signal regulatory protein alpha
NOD: Non-obese-diabetic
HSCs: hematopoietic stem cells
DCs: dendritic cells
i.p.: intrahepatically
RT: room temperature
PBMCs: peripheral blood mononuclear cells
pDCs: plasmacytoid DCs
mDCs: myeloid DCs
p.i.: post-infection
