# Supplementary material for: Long-term leukocyte reconstitution in NSG mice transplanted with human cord blood hematopoietic stem and progenitor cells
Source: BMC Immunol. 2017 May 30;18:28. doi: 10.1186/s12865-017-0209-9 (PMC5450051; doi:10.1186/s12865-017-0209-9)
Supplement: Supplementary file 1 — Antibodies used for flow cytometry. (DOCX 40 kb) [file 12865_2017_209_MOESM1_ESM.docx]

**Supplementary Table 1: Antibodies used for flow cytometry**

|  | Fluorochrome | Clone | Vendor |
| --- | --- | --- | --- |
| Suppl. Fig. 1A : |  |  |  |
| CD45 | PerCP | HI30 | BioLegend |
| CD19 | APC | HIB19 | BioLegend |
| CD14 | PE-Cy7 | HCD14 | BioLegend |
| CD303 | FITC | AC144 | Miltenyi Biotec |
| CD1c | BV 421 | L161 | BioLegend |
| CD141 | PE | M80 | BioLegend |
| Suppl. Fig. 1B |  |  |  |
| CD45 | PerCP | HI30 | BioLegend |
| CD3 | FITC | HIT3a | BD Biosciences |
| CD4 | PE-Cy7 | RPA-T4 | BioLegend |
| CD8 | BV 421 | RPA-T8 | BioLegend |
| NKp46 | PE | 9E2 | BioLegend |
| Suppl. Fig. 3 |  |  |  |
| CD45 | Krome Orange | J.33 | Beckman Coulter |
| CD33 | APC | WM53 | BioLegend |
| Suppl. Fig. 4A |  |  |  |
| CD45 | Krome Orange | J.33 | Beckman Coulter |
| CD3 | BV 421 | UCHT1 | BioLegend |
| CD4 | ECD | SFCI12T4D11 | Beckman Coulter |
| CD8 | APC | RPA-T8 | BD Biosciences |
| CD45RO | PE-Cy7 | UCHL1 | BioLegend |
| CCR7 | PE | G043H7 | BioLegend |
| CD27 | FITC | O323 | BioLegend |
| Suppl. Fig. 4B |  |  |  |
| CD45 | Krome Orange | J.33 | Beckman Coulter |
| CD19 | BV 421 | HIB19 | BD Biosciences |
| CD24 | BV 421 | ML5 | BioLegend |
| CD38 | APC | HIT2 | BD Biosciences |
| Suppl. Fig. 5 |  |  |  |
| CD45 | Krome Orange | J.33 | Beckman Coulter |
| CD34 | APC | 581 | BD Biosciences |
| CD38 | PE-Cy7 | HIT2 | BioLegend |
| CD90 | BV 421 | 5E10 | BioLegend |
