# Supplementary figures and images for: Long-term leukocyte reconstitution in NSG mice transplanted with human cord blood hematopoietic stem and progenitor cells
Source: BMC Immunol. 2017 May 30;18:28. doi: 10.1186/s12865-017-0209-9 (PMC5450051; doi:10.1186/s12865-017-0209-9)

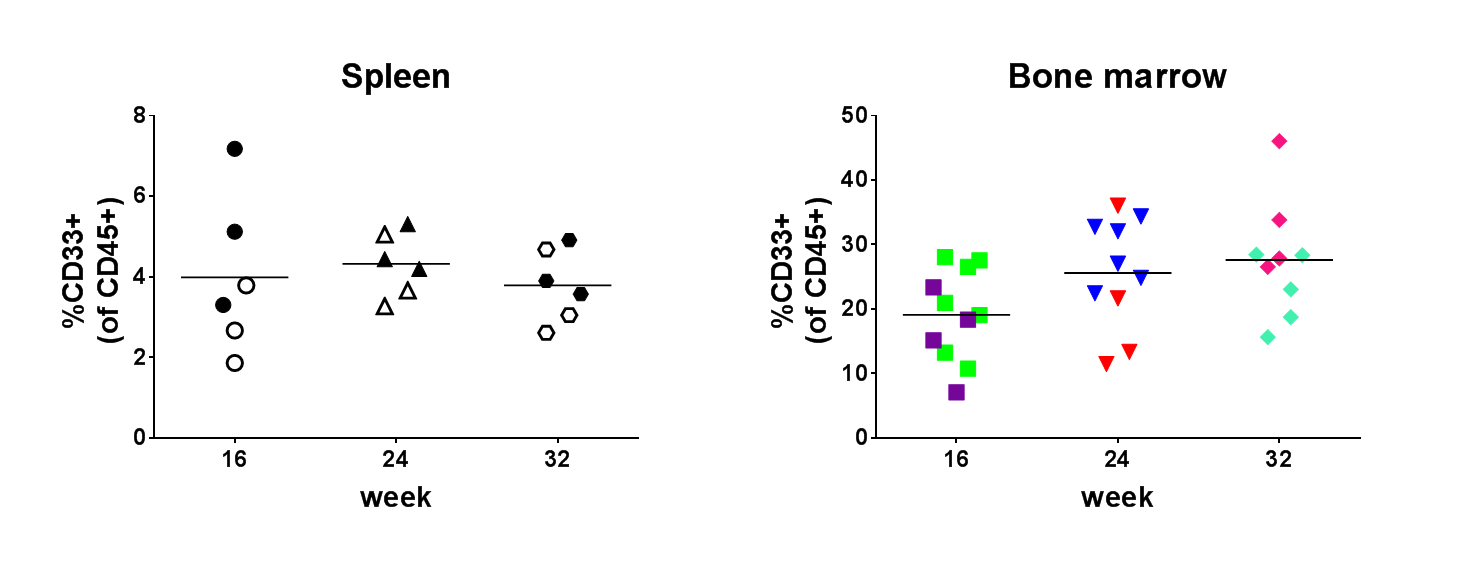

Supplement: Supplementary file 2 — Myeloid cell reconstitution in spleen and bone marrow. Percentage of myeloid cells (CD33+) in spleen (A) and bone marrow (B). Gating strategy is shown in Additional file 4: Figure S4. Each symbol represents an individual mouse; for each time point post-transplantation, data are derived from two different groups of mice, which are indicated by different symbols (spleen: open/closed, bone marrow: different colors); each group was transplanted with cells derived from a different cord blood. (TIF 104 kb) [file 12865_2017_209_MOESM2_ESM.tif]

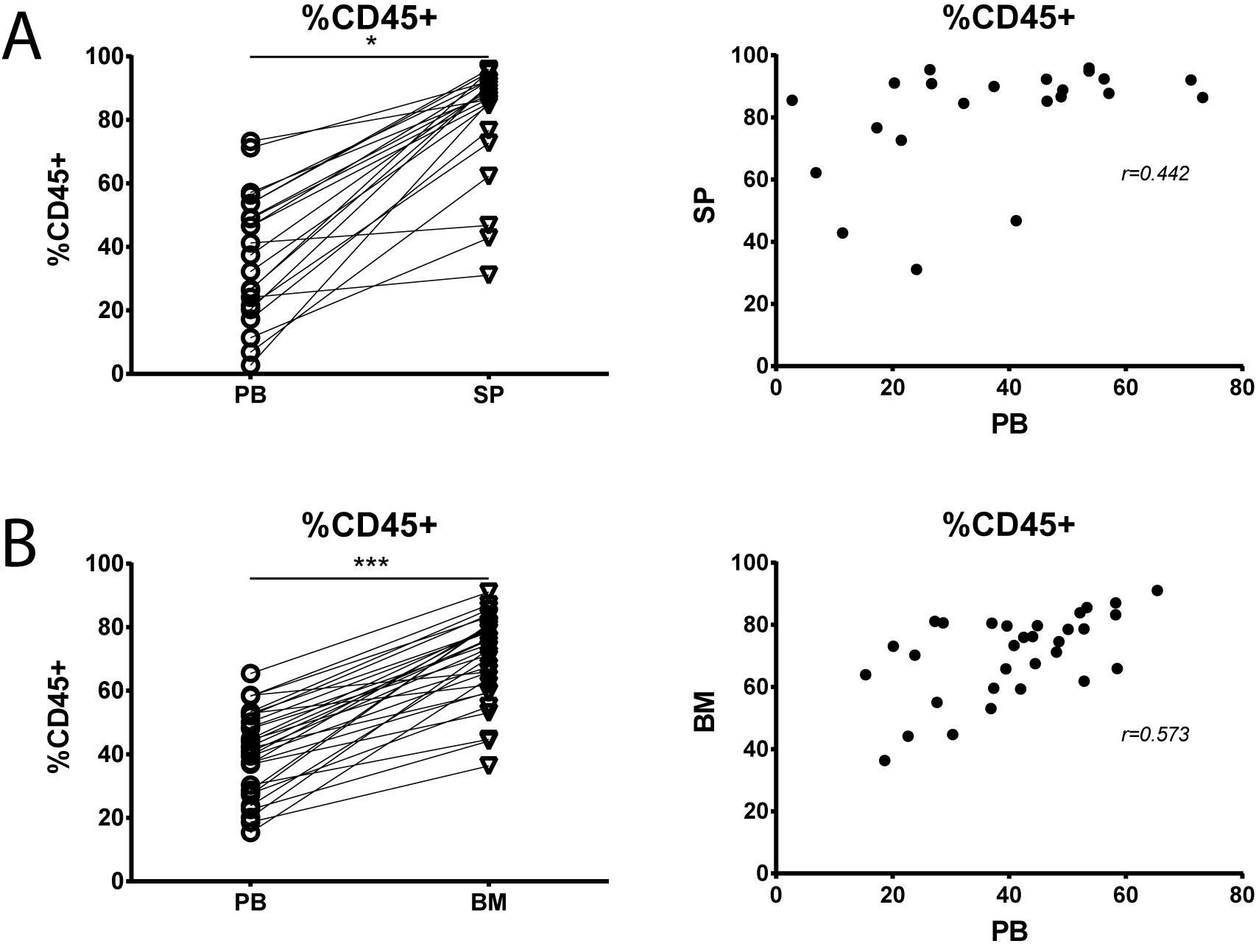

Supplement: Supplementary file 3 — Comparison of human cell chimerism between peripheral blood and spleen or bone marrow. Paired t test (left) and correlation (right) analyses were performed of human cell chimerism in peripheral blood and spleen (A) or bone marrow (B) (TIF 9278 kb) [file 12865_2017_209_MOESM3_ESM.tif]

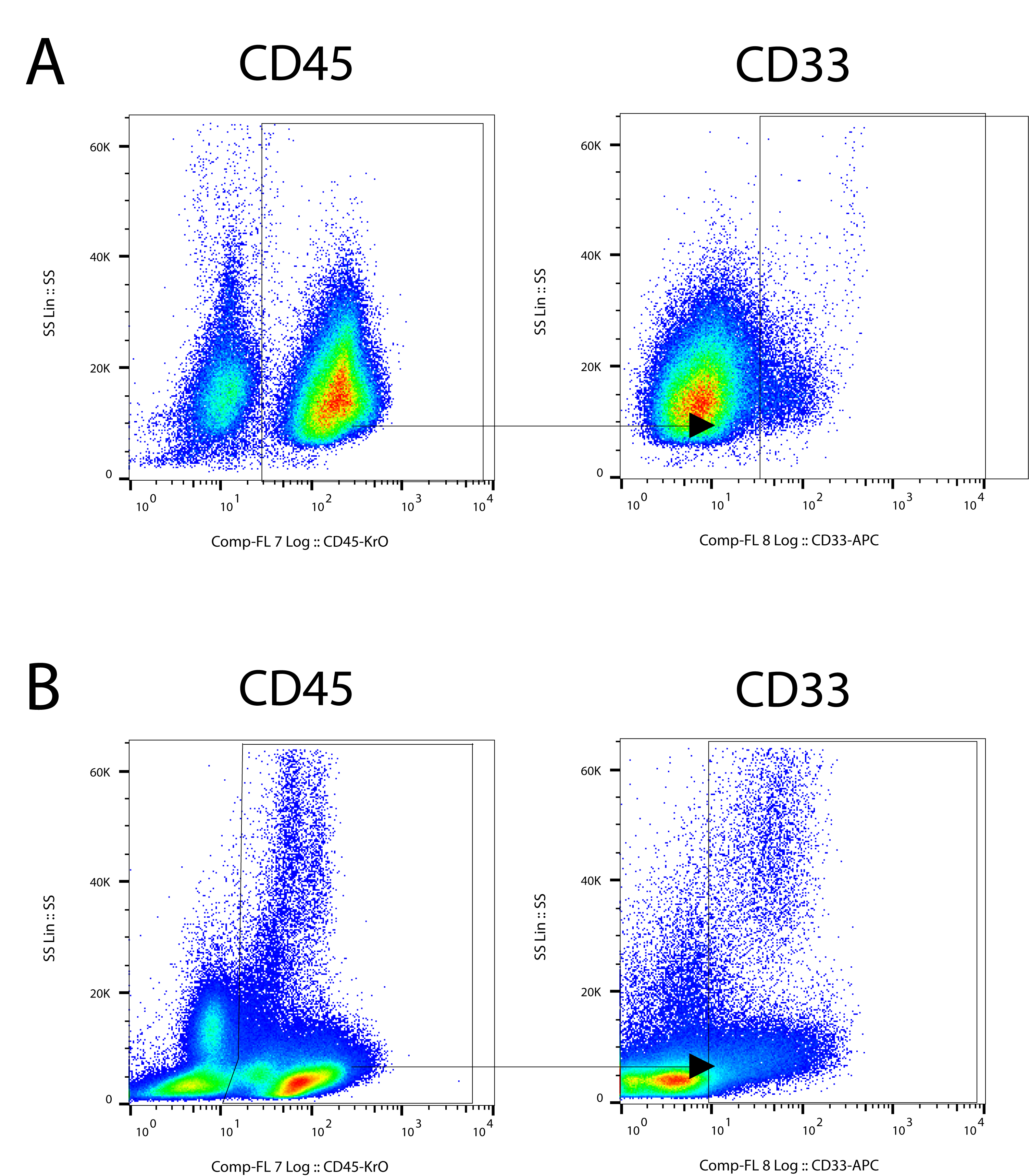

Supplement: Supplementary file 4 — Gating strategy for assessment of myeloid cell reconstitution in spleen and bone marrow. Representative examples for the assessment of myeloid cell (CD33+) reconstitution in spleen (A) and bone marrow (B) are shown. (TIF 30248 kb) [file 12865_2017_209_MOESM4_ESM.tif]

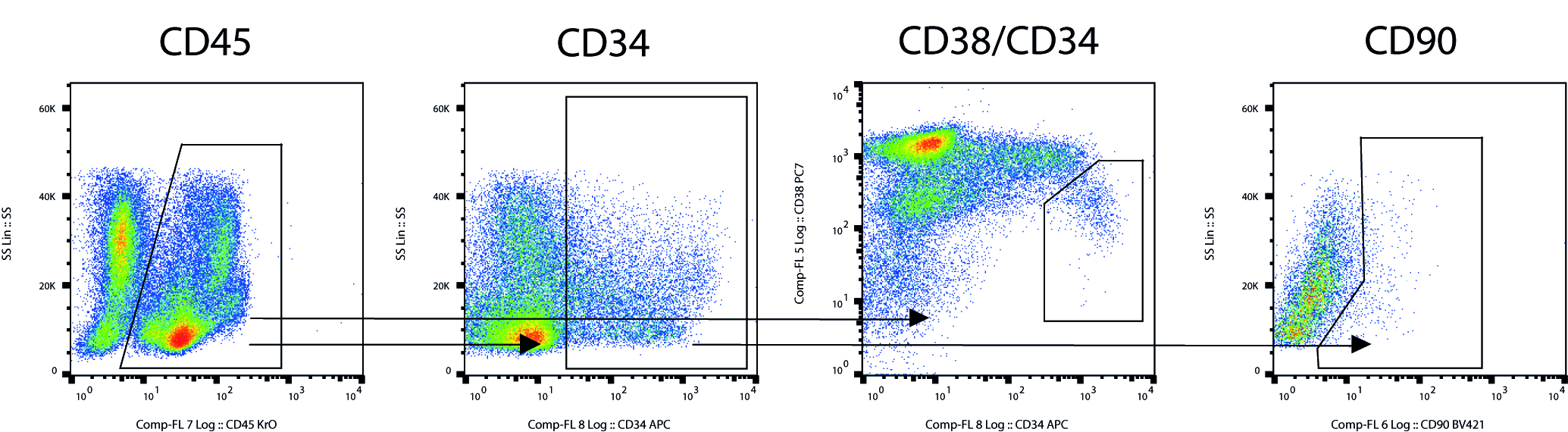

Supplement: Supplementary file 6 — Gating strategy for assessment of engraftment of HSPCs in bone marrow. A representative example for the assessment of engraftment of HSPCs in bone marrow is shown. Percentages of cell populations were determined as follows: Total HSPCs: %CD34+ cells (of CD45+ cells), more primitive cells: %CD38- or CD90+ cells (of CD45 + CD34+ or CD45+ cells). (TIF 5264 kb) [file 12865_2017_209_MOESM6_ESM.tif]

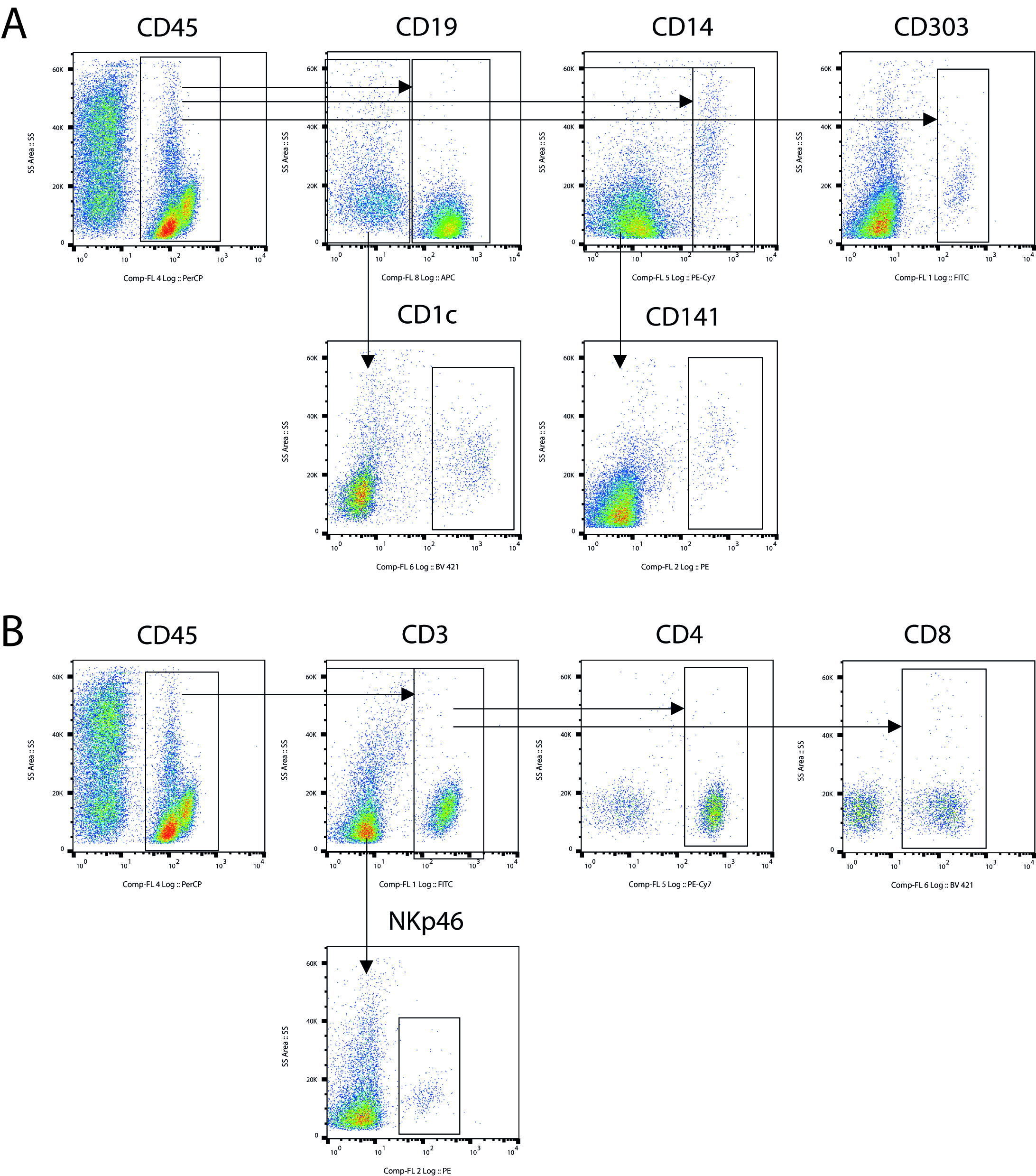

Supplement: Supplementary file 7 — Gating strategy for assessment of human cell chimerism and reconstitution of leukocytes in peripheral blood. A representative example for the assessment of human cell chimerism and leukocyte reconstitution is shown. A: chimerism: %CD45+ cells (of live cells), B cells: %CD19+ cells (of CD45+ cells), monocytes: %CD14+ (of CD45+ cells), pDCs: %CD303+ cells (of CD45+ cells), CD1c + mDCs: %CD19-CD1c + cells (of CD45+ cells), CD141+ mDCs: %CD14-CD141+ cells (of CD45+ cells); B: T cells: %CD3+ cells (of CD45+ cells), CD4 T cells: %CD4+ cells (of CD45 + CD3+ cells), CD8 T cells: %CD8+ cells (of CD45 + CD3+ cells), NK cells: %CD3-NKp46+ (of CD45+ cells). (TIF 21813 kb) [file 12865_2017_209_MOESM7_ESM.tif]
